# Effect of Fenugreek on Hyperglycemia: A Systematic Review and Meta-Analysis

**DOI:** 10.3390/medicina59020248

**Published:** 2023-01-27

**Authors:** Muhammed Shabil, Ganesh Bushi, Pavan Kalyan Bodige, Pavan Sagar Maradi, Bibhu Prasad Patra, Bijaya K. Padhi, Jagdish Khubchandani

**Affiliations:** 1 National Institute of Pharmaceutical Education and Research-Hajipur, Hajipur 844102, India; 2 Department of Community Medicine and School of Public Health, Postgraduate Institute of Medical Education and Research, Chandigarh 160012, India; 3 Department of Public Health, New Mexico State University, Las Cruces, NM 88003, USA

**Keywords:** fenugreek, hypoglycemia, systematic review, meta-analysis

## Abstract

Fenugreek is used for medicinal purposes in various traditions. Some studies have demonstrated that the seeds of this plant may have an anti-diabetic effect by lowering fasting blood sugar levels and improving glucose tolerance. We conducted a systematic review of the hypoglycemic effects of fenugreek. An electronic literature search was carried out in the PubMed, Google Scholar, Scopus, and Cochrane Library databases through 18 November 2022 to find trials that assessed fasting blood glucose, postprandial blood glucose, and HbA1c changes in participants treated with fenugreek and in the control group. The mean difference with 95% confidence intervals (CI) was calculated to represent the analysis. Fourteen trials, consisting of 894 participants, were included in the meta-analysis. The results showed a reduction in fasting blood glucose levels (MD: 3.70, 95% CI of −27.02, 19.62; *p* = 0.76), postprandial blood glucose (MD: −10.61, 95% CI of −68.48, 47.26; *p* = 0.72), and HbA1c (MD: −0.88, 95% CI −1.49, −0.27; *p* = 0.00) with fenugreek consumption. While this review and included trials that found beneficial effects of fenugreek consumption on glycemic control, the quality and heterogeneity of studies remain a concern. Given the wider availability and lower cost of fenugreek, rigorous double-blinded randomized controlled trials should be conducted with fenugreek to understand its true potential as a diabetes control herbal agent.

## 1. Introduction

Diabetes affects half a billion people globally and is expected to rise by 25% in 2030 and by 51% in 2045 [1]. Type 2 diabetes (T2DM) has increased rapidly in developing nations, with its prevalence varying depending on the sociodemographic characteristics of population groups (e.g., income, education, employment, level of urbanization, etc.). Certain racial and ethnic groups (e.g., South Asians and African natives) experience an earlier onset of diabetes, exhibit noticeable abdominal obesity, live with chronic lifestyle behavioral risks, and experience a faster progression from prediabetes to diabetes [2].

Fenugreek (also known as Trigonella foenum-graecum of Fabaceae family) is used widely over the world, particularly in India, Egypt, China, and Middle Eastern nations, for both cooking purposes and the treatment of T2DM [3,4,5]. For its effectiveness in the diabetic population, several mechanisms have been proposed. Alkaloids such as trigonelline and Fenugrecin present in fenugreek have been shown to have hypoglycemic activity, whereas soluble fibers such as glucomannan fiber and 4-hydroxyisoleucine (4-OH Ile) amino acids stimulate the production of insulin from the pancreas [6]. Furthermore, a clinical study showed that fenugreek’s antidiabetic effect was accomplished by increasing insulin sensitivity [6]. In contrast, some studies have found that fenugreek seeds may decrease postprandial insulin and glucose levels (PPG) [7,8,9,10]. Additionally, a few longer-term trials with fenugreek have also demonstrated decreases in fasting plasma glucose (FPG) and PPG as well as glycated hemoglobin (HbA1c) [10,11,12], whereas some studies did not yield positive results [13,14]. Given the conflicting evidence from multiple studies, we conducted an updated systematic review and meta-analysis on the effects of fenugreek on glycemia by including randomized controlled trials (RCTs) only.

## 2. Materials and Methods

The Cochrane Handbook’s recommendations were followed in the planned methodology used to conduct this systematic review and meta-analysis [15]. According to the Preferred Reporting Items for Systematic Reviews and Meta-Analyses (PRISMA) statement, the findings were reported [16]. This systematic review was registered in the International Prospective Register of Systematic Reviews (PROSPERO) under registration number CRD42022354099.

### 2.1. Search Strategy

An electronic literature search was carried out in databases: PubMed, Google Scholar, Scopus, ClinicalTrials.gov, and the Cochrane Library through 9 November 2022, using keywords and MeSH terms. The following keywords were used in the search strategy: ((Fenugreek OR Trigonella) AND (Intervention OR “controlled trial” OR randomized OR random OR randomly OR placebo OR “clinical trial” OR Trial OR “randomized clinical trial” OR RCT OR trial OR trials “Cross-Over Studies” OR “Cross-Over” OR “Cross-Over Study” OR parallel OR “parallel study” OR “parallel trial”) AND (“diabetes” OR “type 2 diabetes mellitus” OR “T2DM” OR “type 2 diabetes” OR “Glucose Intolerance”. OR “glycemia” “Hyperglycemia” “T2D” OR “prediabetes”)). The search was performed without any language or year restriction. The search results were downloaded into the EndNote software.

### 2.2. Inclusion and Exclusion Criteria

To determine the effects of fenugreek on glycemia, we included studies in which subjects were healthy, had T2DM, or were prediabetic. The intervention was limited to the consumption of fenugreek alone, in any form or at any dose. Only randomized controlled trials with at least 7 days of intervention were included. The outcomes of interest were FBG, PPG, HbA1c levels. Studies without a control group and those that did not report baseline values were not included. We also excluded studies that used combinations of other herbs with fenugreek for the intervention. Narrative reviews, observational studies, animal studies, case reports, and letters were also excluded.

### 2.3. Study Selection

The screening of studies and selection processes were carried out according to PRISMA suggestions. The search results from the databases were downloaded into Endnote and uploaded to the Rayyan software for primary screening. Initial screening was performed by two independent reviewers (MS and GB) by reading the titles/abstracts. Disagreements were resolved by discussion with other reviewers (PKB and PSM). After the initial screening, a thorough full-text reading was performed to identify potential studies.

### 2.4. Data Extraction and Quality Assessment

Data extraction was performed from the included studies by using a predetermined standard form. The author’s name, publication country, year, sample size, study design, subject characteristics, combined medications, fenugreek form and dose, intervention duration, and outcomes of interest were extracted from each article.

Using the “Risk of Bias” assessment tool developed by the Cochrane Collaboration, we evaluated the risk of bias for each included study. The following specific domains of bias, including selection, performance, detection, attrition, reporting, and other sources of bias, were covered by the risk of bias evaluation standards of the Cochrane Handbook [17]. Each item was assessed and given “yes”, “no”, or “unclear” for “low risk”, “high risk”, or “unclear risk” of bias accordingly.

### 2.5. Statistical Analysis

The STATA version 16 was used to evaluate the data and to create forest plots. A random-effects model was used to consider the heterogeneity among the trials [18]. Mean differences and 95% confidence intervals (CI) were calculated for the outcome variables, and *p* ≤ 0.05 was set as the significance level. Tests for heterogeneity across included studies were assessed using the tau squared (τ^2^) and I -squared statistics [18]. A meta-analysis was performed if the outcome of interest was available for at least three studies. A funnel plot was used to determine the presence of publication bias by visual inspection. Meta-regression was performed to find out the effect of any variable, and sensitivity analysis was also performed to determine the effect of individual studies on results.

## 3. Results

### 3.1. Characteristics of Included Studies

**Figure 1:**
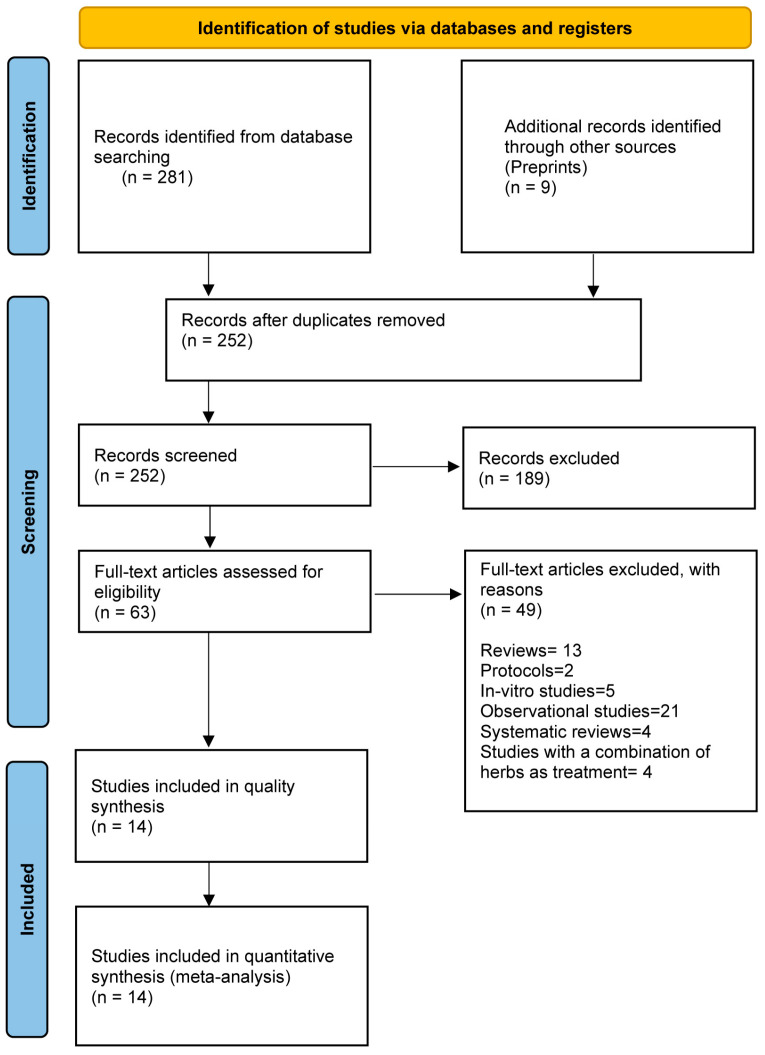
PRISMA flowchart showing the search and screening process.

The literature search results and the selection process are presented in Figure 1. Overall, 290 records were identified in the literature search of all databases, of which 38 were duplicates. A total of 252 articles were subjected to primary screening. Thirteen reviews, twenty-one observational studies, five in vitro studies, two protocols, four studies with a combination of herb as treatment, and four systematic reviews were excluded in full-text reading. Six studies were finally included after full-text reading.

### 3.2. Characteristics of Included Studies

A total of 894 subjects participated in the trials (Table 1). Among the included fourteen trials, eight were conducted in India, three in Iran, and one each in China, France, and Egypt. All RCTs had a parallel design. Twelve studies included patients with T2DM. One study included overweight and healthy individuals, while another study included pre-diabetic participants. The dose of fenugreek administered varied across trials. Fenugreek was administered once, twice, or thrice a day. Seven studies had no placebos in the control arm. Thirteen studies reported fasting blood glucose levels as an outcome. Six studies reported postprandial glucose levels, and seven studies reported HbA1c levels. Two trials were conducted using the FENFURO capsule as an investigational product. The duration of the study ranged from one week to three years. 

### 3.3. Risk of Bias

**Figure 2:**
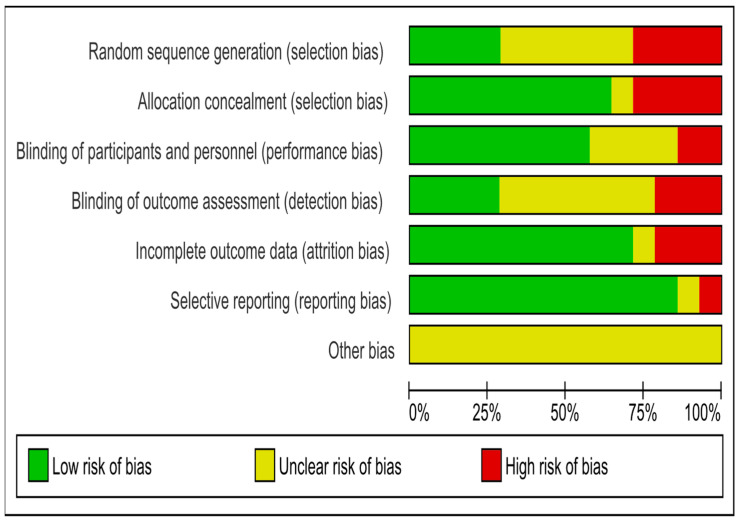
Risk of bias of included trials.

Of the 14 trials, only 4 were of high quality. Ten studies were RCTs. Only four studies were clear regarding the method of randomization. Four studies had selection bias and six were unclear regarding randomization. Nine studies reported on allocation concealment. Four trials had selection bias, and one study had unclear allocation concealment. Eight studies reported blinding of the participants and personnel. Two studies had a performance bias, and four were unclear. Only four studies mentioned blinding of outcome assessment, seven were unclear, and three had detection bias. Four studies did not mention dropouts, and selective reporting was detected in only one study. The risk of bias assessment for all included RCTs is shown in Figure 2.

### 3.4. Effect of Fenugreek on Fasting Blood Glucose Level

**Figure 3:**
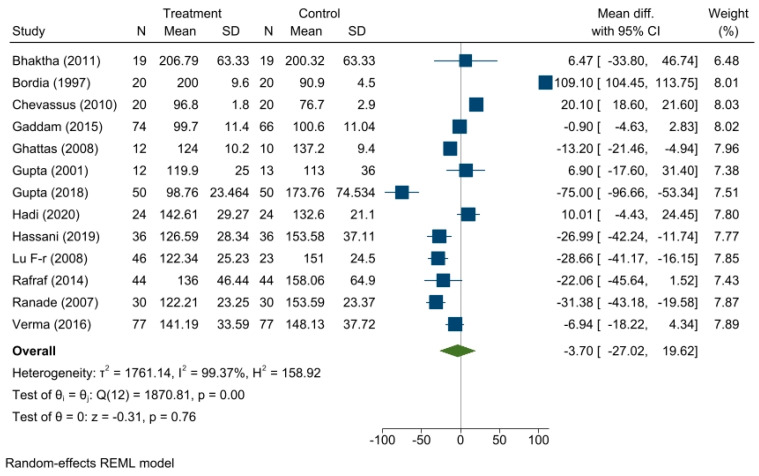
Forest plot showing the effects of fenugreek on fasting blood glucose level.

A reduction in FBG levels was found in the treatment group compared to the control group but was not significant, with a summary effect measure of a mean difference of −3.70, 95% CI of −27.02, 19.62; *p* = 0.76. Heterogeneity among the studies was found to be highly significant (τ^2^ = 1761.14, I^2^ = 99.37%). Forest plot is shown in Figure 3.

### 3.5. Effect of Fenugreek on Postprandial Glucose Level

**Figure 4:**
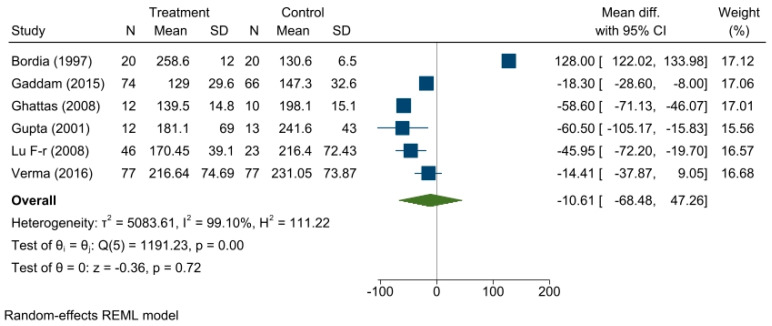
Forest plot showing the effect of fenugreek on postprandial blood glucose level.

From the six trials that reported postprandial glucose levels, a reduction in postprandial glucose levels in the treatment group was found compared to the control group with a summary effect measure of a mean difference of −10.61, 95% CI (−68.48, −47.26; *p* = 0.72). Heterogeneity among the studies was found (τ^2^ = 5083.61, I^2^ = 99.10%). The forest plot is shown in Figure 4.

### 3.6. Effect of Fenugreek on HbA1c

**Figure 5:**
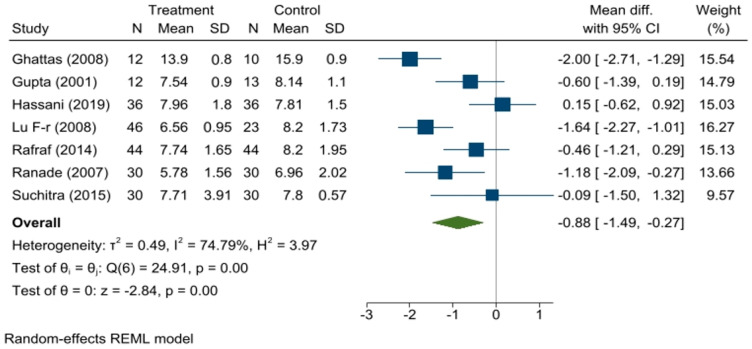
Forest plot showing the effect of fenugreek on HbA1c levels.

A significant reduction in HbA1c levels in the treatment group was found compared to that in the control arm with a summary effect measure of a mean difference of −0.88, 95% CI (−1.49, −0.27; *p* = 0.00). Heterogeneity among the studies was found to be significant (τ^2^ = 0.49, I^2^ = 74.79%, *p* = 0.00). The forest plot is shown in Figure 5.

### 3.7. Publication Bias

**Figure 6:**
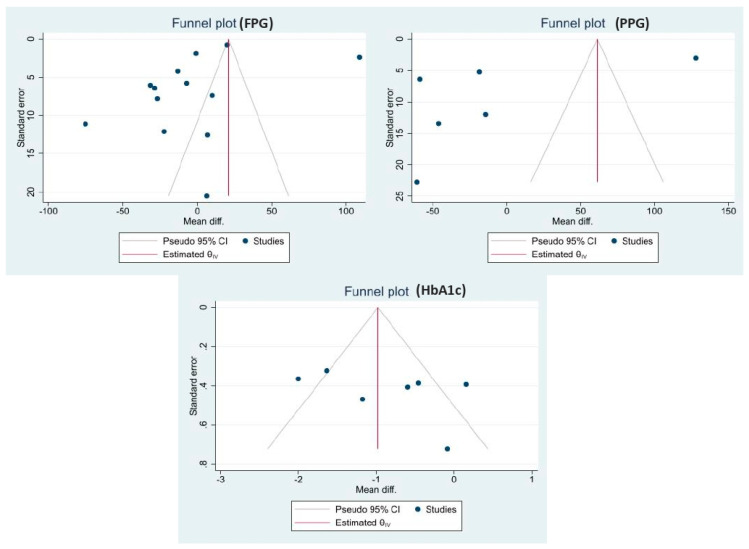
Funnel plots showing publication bias.

Publication bias was identified by funnel plots (Figure 6). Dissymmetry was found in the visual inspection of the funnel plots indicating the possibility of publication bias. Regression-based Egger test found no small study effects (*p* = 0.237).

### 3.8. Meta-Regression

**Figure 7:**
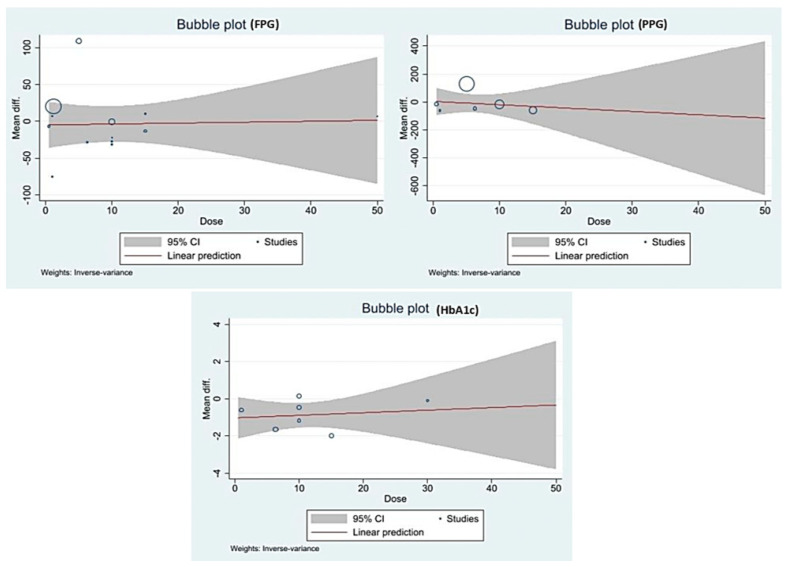
Bubble plots showing the association of dose with the mean difference.

We performed univariate and multivariate meta-regression to determine the effects of dose, treatment duration, study design, and total sample size on the effect size of FPG (Table 2). The multivariate regression analysis indicates that the study design (RCT) significantly contributing the reduction of FPG β-80.814 (95%CI: −141.917, −19.712). A bubble plot showing the dose versus mean difference is given in Figure 7. The flat regression line indicates no association between the dose and the effect size of FPG, PPG, and HbA1c. 

### 3.9. Subgroup Analsysis

**Figure A1:**
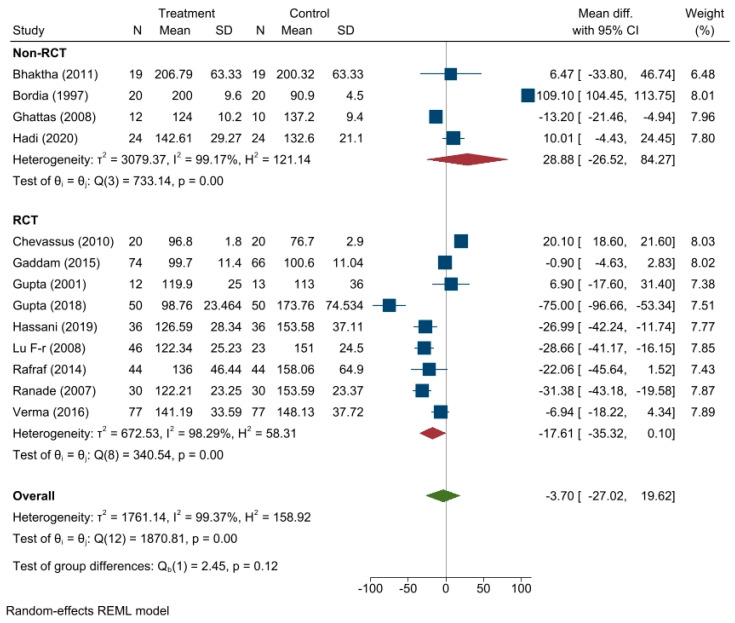
Forest plot showing subgroup analysis based on RCT/non RCT for effect of fenugreek on FPG.

**Figure A2:**
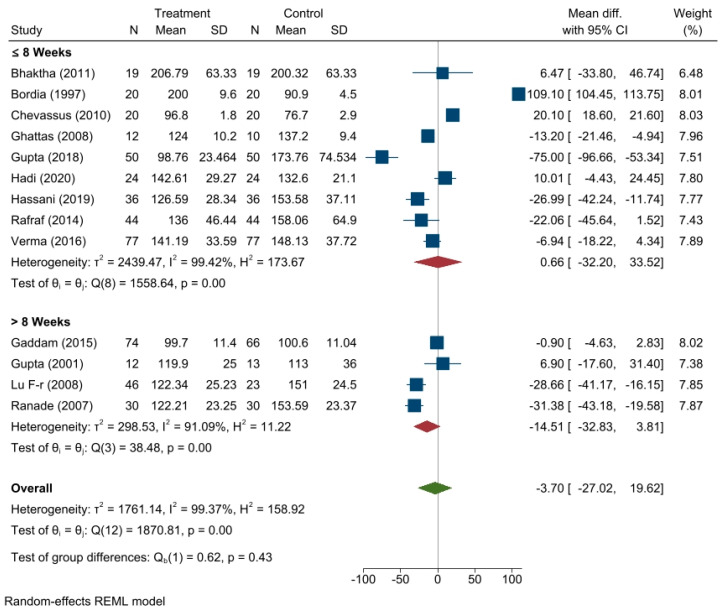
Forest plot showing subgroup analysis based on treatment duration for effect of fenugreek on FPG.

Subgroup analysis was performed based on RCT/non-RCT and treatment duration for the effect of fenugreek on the FPG level. Four studies were non-RCTs and nine were RCTs. A pooled effect estimate of 28.88 mg/dL, 95% CI (26.52, 84.27) has been found for non RCTs and −17.61 mg/dL, 95% CI (−35.32, 0.10) obtained from RCTs (Figure A1). Nine studies had a treatment duration of ≤8 weeks and four studies had a treatment duration of >8 weeks. A pooled effect estimate of 0.66 mg/dL, 95% CI (−32.20, 33.52) has been found for treatment duration ≤8 weeks and −14.51 mg/dL, 95% CI (−32.83, 3.81) obtained from studies of treatment duration > 8 weeks (Figure A2).

### 3.10. Sensitivity Analysis

**Figure A3:**
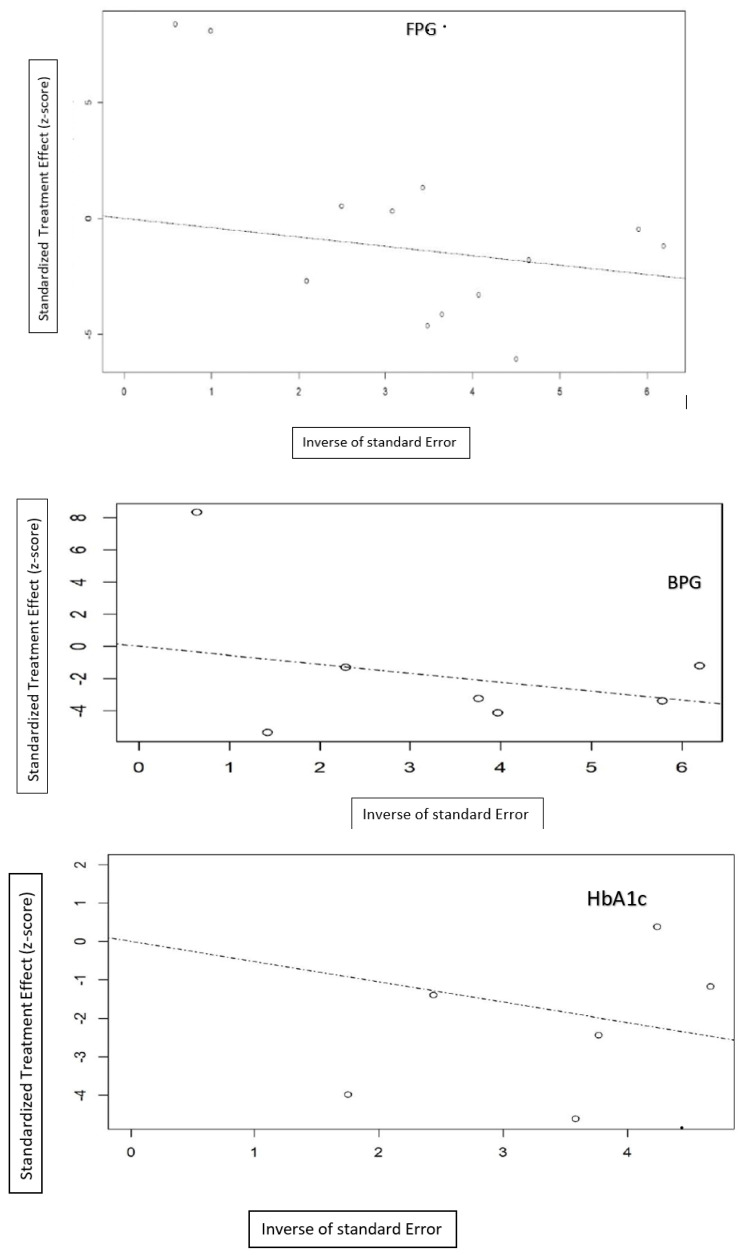
Radial plots.

We also conducted sensitivity analyses of the effects of fenugreek on FBG, PPG, and HbA1c concentrations, omitting one study at a time. No significant change was observed for any study on FPG level which ranged from −13.41 mg/dL (95% CI: −27.65, −10.83) to −2.14 (95% CI: −20.04, −24.33). As for the PPG level, omitting the study conducted by Bordia et al. [24] resulted in significant reduction of −37.45 mg/dL (95% CI: −57.38, −17.53), *p* = 0.00. None of the individual trials dramatically influenced pooled effect estimates of HbA1c, which ranged from and from −1.08% (95% CI: −1.66, −0.51) to −0.69 (95% CI: −1.26 to −0.11) for HbA1c. Radial plots are given in Figure A3.

## 4. Discussion

In this meta-analysis of 14 trials, fenugreek seed consumption reduced the FPG by 3.7 mg/dL, PPG by 10.61 mg/dL, and HbA1c by 0.88%. However, only reduction in HbA1c was statistically significant. However, sensitivity analysis showed a significant reduction in PPG levels by −37.45 mg/dL when the trial conducted by Bordia et al. [26] was omitted. This trial was of poor quality. Substantial heterogeneity was observed among the studies. Differences in diabetes status, daily dose of fenugreek, and type of fenugreek extract might have contributed to heterogeneity. Subgroup analysis revealed that RCTs and treatment duration more than 8 weeks provided better reduction in the FPG levels. Meta regression also revealed study design (RCT) was affecting reduction of FPG. The dietary habits of the participants were not considered in many trials. The type and dosage of antidiabetic drugs taken by the participants were unclear in some trials. How long the subjects were diagnosed with T2DM and when they started to take antidiabetic medication was also not mentioned in some studies. These factors may explain the heterogeneity. Two trials involved FENFURO, a proprietary product with good glycemic effects. Fenugreek was administered at different doses across studies. The quality and purity of the composition may have varied, thereby influencing the results. The physical activity of the study participants across the groups might also have affected the results. Our findings are in line with those of nearly a decade old reviews of fenugreek consumption in relation to blood sugar [4,31]. Our analysis provides a major update on the association between fenugreek and blood glucose by including the most rigorous trials to date.

According to recent biochemical studies, fenugreek seeds have anti-diabetic properties by prolonging gastric emptying time and lowering glucose uptake in the small intestine because of their high fiber content, thereby slowing down carbohydrate metabolism and lowering blood glucose levels. Additionally, there is a restoration of pancreatic cells by safeguarding β-cells [32,33] and an increment in serum insulin levels by stimulation of islet cell regeneration or insulin release from pre-existing islet cells. Fenugreek also stimulates glycogen synthase activity and promotes the production of liver and muscle glycogen [34]. This promotes the regeneration of depleted glycogen, decreases pro-inflammatory cytokines and pancreatic enzymes, and adjusts the serum lipid profiles and the activity of insulin-sensitive carbohydrate metabolic enzymes [34]. Fenugreek may increase insulin sensitivity by enhancing insulin action at the cellular level, lowering HbA1c levels by using glucose in the peripheral tissues and maintaining blood glucose levels [32,33,34,35]. Not only does fenugreek affect blood sugar, but multiple reviews also suggest a role in the improvement of lipid profiles, making it suitable for metabolic disorders [4,9,24,35,36]. It could be possible that there are interrelated mechanisms of action of fenugreek where its biochemical properties could be related to the metabolism of sugar, lipid, or both [35,36].

Fenugreek is available at a low cost in countries across the world and is used for a variety of purposes. Additionally, fenugreek has no known side effects; therefore, it is generally safe to use in patients with diabetes or lipid disorders. Fenugreek can also be used in combination with other herbal medications or as a complementary treatment along with other medications and lifestyle modifications in patients with diabetes or lipid disorders. Given the results of this study and prior findings of fenugreek’s effect on metabolism, there is a potential for its wider consumption and usage if additional studies continue to help confirm the effects and improve our understanding of its biochemical mechanisms of action. The trials included in the systematic review had biases. Further studies related to trials on fenugreek should follow standard guidelines to produce high-quality confirmatory results and thereby overcome the limitations of existing studies. Given the lower quality of existing trials and the possibility of publication bias, more double-blinded RCTs should be performed following strict guidelines for herbal interventions. Fenugreek must be composition-tested, and the optimum dose/frequency of fenugreek per day should be determined and standardized to ensure robust scientific evidence on this low-cost measure for millions of individuals struggling with diabetes and other metabolic disorders. 

**Figure A4:**
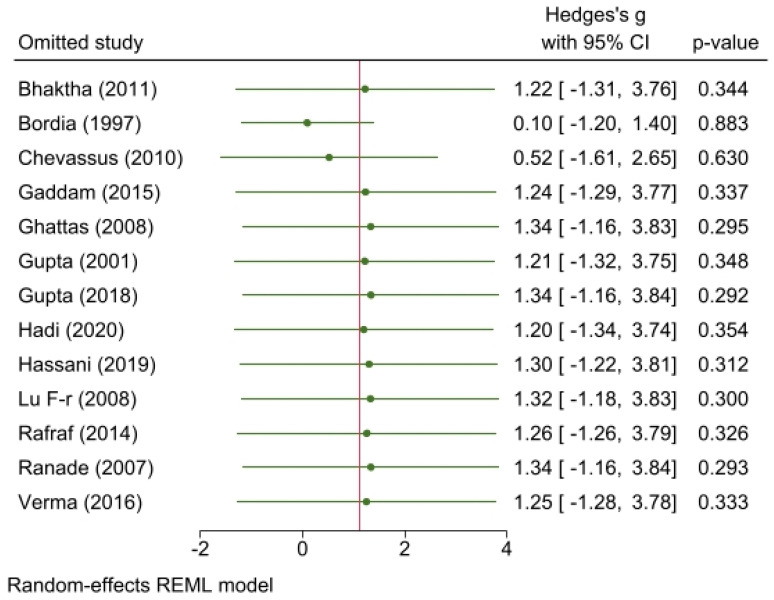
Leave one out analysis.

**Figure A5:**
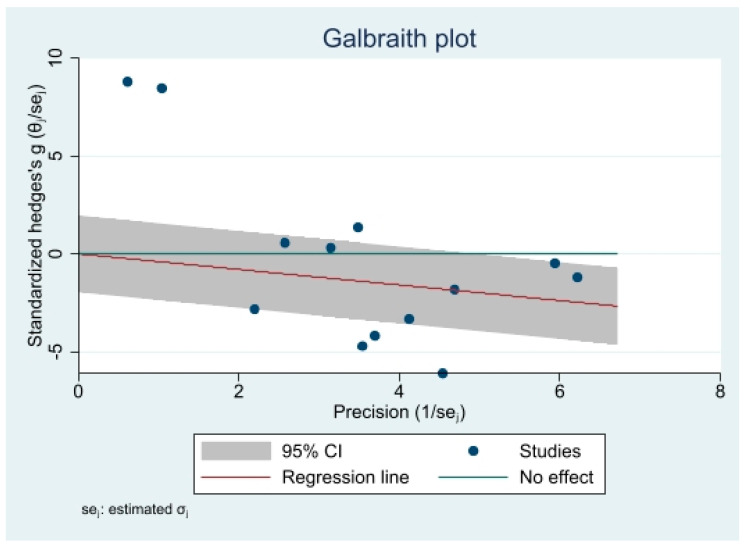
Galbraith plot.

We could perform meta-regression and sensitivity analysis (Figure A4 and Figure A5), but our study is not free from limitations. Many trials were of poor quality, and there was a substantial publication bias. Subgroup analysis failed to address the heterogeneity. Although a broad and thorough search was conducted, language bias was unavoidable. Additionally, fenugreek is an herbal supplement, and its crude form can vary in terms of quality. The details of the diabetes medications used were unclear in many of the studies. Despite these limitations, our review is the most comprehensive and inclusive to date showing the promise of fenugreek as a supplement. 

## Figures and Tables

**Table 1:** Characteristics of trials included for review.

Author	Country, Year	Design	Age	Population	Subjects (*n*)	Medications	FENUGREEK FORM	Daily Dose	Treatment Duration	Control Group	Outcomes of Interest
Verma N [19]	India, 2016	Parallel	25–60	T2DM < 5 years	154	Metformin	FENFURO capsule	0.5 g	4 weeks	Placebo	FBG, PPG
Hadi A [20]	Iran, 2020	Parallel	30–65	T2DM	48	Anti-diabetic drugs	Powder	15 g	8 weeks	No placebo	FBG
Bhaktha G [21]	India, 2011	Parallel	30–45	New diagnosed T2DM	38	N/A	Powder	50 g	8 weeks	No placebo	FBG
Gupta R [22]	India, 2018	Parallel	28–65	T2DM	100	Anti-diabetic drugs	FENFURO capsule	1 g	12 weeks	No placebo	FPG, HbA1c
Lu F-r [11]	China, 2008	Parallel	54.26	T2DM poorly controlled	69	Sulphonyl urea	Capsule	6.3 g	12 weeks	Chinese yam as placebo	FBG, PPG, HbA1c
Gupta A [23]	India, 2001	Parallel	51	NIDDM	25	Sulphonyl urea/biguanide	Capsule extract	1 g	8 weeks	Placebo is given	FPG, PPG, HbA1c
A. Bordia [24]	India, 1997	Parallel	N/A	T2DM	40	N/A	Capsule	5 g	4 weeks	Placebo is given	FBG, PPG,
Rafraf M [25]	Iran, 2014	Parallel	40.535	T2DM	44	Metformin/ Glibemclamide	Powder	10 g	8 weeks	Placebo is given	FBG, HbA1c
Chevassus H [26]	France, 2010	Parallel	38.0	Healthy overweight	39	N/A	Coated tablet	1176 mg	6 weeks	Placebo is given	FBG
Suchitra M [27]	India, 2015	Parallel	50.2	T2DM	60	Oral hypoglycemic agent	Fenugreek seeds	30 g	8 weeks	No placebo	HbA1c
Gaddam A [28]	India, 2015	Parallel	30–70	Pre-diabetic	79	N/A	Powder	10 g	3 years	No placebo	FBG, PPG
Ghattas LA [29]	Egypt, 2008	Parallel	43–64	T2DM	22	N/A	Powder	15 g	1 week	No placebo	HbA1c, FBG, PPG
Ranade M [6]	India, 2007	Parallel	46.22–48	T2DM	60	Anti-diabetic medication	Fenugreek seed, water soaked	10 g	6 months	No placebo	FBG, HbA1c
Hassani SS [30]	Iran, 2019	Parallel	51.27	T2DM	72	N/A	Powder	10 g	8 weeks	Wheat flour placebo	FBG, HbA1c

Abbreviations: T2DM: Type 2 diabetes mellitus, FBG: Fasting blood glucose, PPG: Post prandial blood glucose, HbA1c: Glycated hemoglobin, NIDDM: Non-insulin-dependent diabetes mellitus, N/A: Not available.

**Table 2:** Meta-regression for the factors contributing the effect size of FPG (*n* = 13).

Variables	β (95%CI)		*p*-Value
	Unadjusted	Adjusted	
Treatment Dose (mg)	0.125 (−1.938, 2.190)	−1.424 (−3.665, 0.817)	0.213
Treatment Duration (Weeks)	−0.030 (−0.686, 0.625)	0.181 (−0.410, 0.772)	0.548
Study design (RCT)	−47.680 (−92.620, −2.740)	−80.814 (−141.917, −19.712)	0.010
Total Sample	−0.030 (−0.733, 0.672)	0.273 (−0.411, 0.959)	0.433

## Data Availability

Not applicable.
